# High attack frequency in patients with angioedema due to C1-inhibitor deficiency is a major determinant in switching to home therapy: a real-life observational study

**DOI:** 10.1186/s13023-016-0518-8

**Published:** 2016-09-29

**Authors:** Veronica Squeglia, Alessandro Barbarino, Maria Bova, Carmela Gravante, Angelica Petraroli, Giuseppe Spadaro, Massimo Triggiani, Arturo Genovese, Gianni Marone

**Affiliations:** 1 Dipartimento di Science Mediche Traslazionali, Università degli Studi di Napoli Federico II, Via S. Pansini 5, 80131 Naples, Italy; 2 Department of Public Health, Università degli Studi di Napoli Federico II, Naples, Italy; 3 Department of Allergy and Clinical Immunology, University of Salerno, Salerno, Italy

**Keywords:** C1-inhibitor deficiency, Hereditary angioedema, C1-inhibitor concentrates, Icatibant, Home therapy, Self-administration, Attack frequency

## Abstract

**Background:**

Hereditary angioedema with C1-inhibitor deficiency (C1-INH-HAE) is characterized by recurrent attacks of swelling that affect various body sites. Such attacks are a frequent cause of visits to the emergency department and are often treated in the hospital. In recent years, self-administration of C1-inhibitor (C1-INH) concentrates at home has become an increasingly used option, with a positive impact on patient outcomes and quality of life.

**Methods:**

This was an observational study of 6 months’ duration in 56 patients with C1-INH-HAE referred to a HAE center in southern Italy. The patients received three types of treatment for their swelling attacks: C1-INH concentrates administered at home (*n* = 25); icatibant administered at home (*n* = 12); and C1-INH concentrates administered in the hospital (*n* = 19). The objectives of this observational study were to compare therapy compliance (defined as the proportion of treated attacks) and quality of life in home- and hospital-treated patients, and to identify factors associated with the decision to use home therapy.

**Results:**

Overall, 918 attacks were reported over 6 months, of which 544 (59.2 %) were treated. Total number of reported attacks and the mean (±SD) number of attacks per patient, respectively, in the three groups were: 611 and 24.4 (±26.1) for home-based C1-INH; 191 and 15.9 (±12.0) for home-based icatibant; 166 and 6.1 (±6.5) for hospital-based C1-INH. Differences in attack frequency between home- and hospital-based treatments were statistically significant (*p* = 0.002), while patient demographic characteristics and the disease severity score did not correlate with the use of home therapy. Compliance with therapy was significantly better with home-based therapy (71.2 % of treated attacks with C1-INH and 44.0 % with icatibant) than with hospital-based therapy (21.6 %, *p* = 0.003). Quality of life showed an opposite trend, with patients on hospital-based treatment reporting the highest quality of life.

**Conclusions:**

Home-based therapy was associated with better compliance compared with hospital-based therapy. The choice to adopt home-based therapy appeared to correlate with a high attack frequency. Home-based therapy is a valid treatment option for patients with C1-INH-HAE and should be offered to all such patients, and especially to those with high attack frequency.

## Background

Hereditary angioedema with C1-inhibitor deficiency (C1-INH-HAE) is a rare disease marked by recurrent attacks of swelling that affect various body sites including the gastrointestinal tract, the extremities, the face, the larynx, and the urogenital region [1, 2]. Deficiency in C1-esterase inhibitor (C1-INH) results in the overproduction of bradykinin and activation of bradykinin B2 receptors leading to increased vascular permeability and edema [3].

Severity and frequency of the attacks and the body site affected are unpredictable. HAE attacks can be associated with severe pain and can be life-threatening when the upper airways are involved, due to the risk of asphyxiation. They are a frequent cause of visits to the emergency department and, until recently, HAE treatment was predominantly hospital-based [4, 5]. In recent years, home-based therapy, involving self-administration of therapy or administration by a family member, has been increasingly offered to HAE patients [6]. The evidence suggests that this treatment option may reduce the burden of HAE and improve patient quality of life (QoL) [7, 8]. Home administration of on-demand treatment usually results in a shorter time from the onset of attack to the initiation of treatment, shorter attack duration, and less severe attacks [9–14]. In addition, several studies have shown that home administration of HAE therapies is safe, with a low incidence of adverse events [10, 12, 15, 16]. According to current international guidelines for the treatment of HAE, home-based therapy should be the preferred strategy whenever possible [17–20]. Treatments licensed in Europe for self-administration include plasma-derived (pd) C1-INH concentrates (Berinert^®^, Cinryze^®^) and icatibant (Firazyr^®^), a bradykinin B2 receptor antagonist.

We recently published the results from our experience in teaching pdC1-INH self-administration to a group of patients with C1-INH-HAE [21]. The self-injection training course was proposed to all patients referred to our center; about 40 % attended. Approximately half of the patients who attended the training course decided to switch to home-based therapy. To improve the rate of patients practicing self-therapy and to optimize the selection of patients best suited for this treatment option, we set out to investigate what might affect patients’ choices. We report here the results of an observational study involving all the patients referring to our center and receiving three different types of treatment for their acute attacks, namely home-based pdC1-INH, self-injection of icatibant, or hospital-based pdC1-INH. This study aimed to compare demographic and clinical characteristics, adherence to treatment, and quality of life in patients treated at home versus (vs.) patients treated in the hospital, with the ultimate goal of identifying factors predictive of the choice to initiate home-based therapy.

## Methods

### Study design and patients

This was an observational study of 6 months’ duration, and included all patients with a diagnosis of C1-INH-HAE (type I and type II) in treatment at an HAE referral center in southern Italy, between August 2014 and January 2015. The patients were divided into three groups according to the type of treatment they were receiving for their acute attacks, namely: home-based therapy with C1-INH concentrate (Berinert^®^, CSL Behring; or Cinryze^®^, Shire; administered by intravenous infusion); home-based therapy with icatibant (Firazyr^®^, Shire; administered by subcutaneous injection); hospital-based therapy with C1-INH concentrate (Berinert^®^). In all groups, treatments were administered according to the manufacturer’s indications and local guidelines. The objectives of the study were: 1) to compare compliance with therapy and quality of life in patients treated at home vs. hospital at the end of the 6 month-observation period and; 2) to identify factors associated with the decision to adopt home therapy. The study was approved by the local institutional review board (Comitato Etico Università “Federico II”, Naples, Italy). All patients, or parents/legal guardians for minors, gave their informed consent to data collection and analysis.

### Self-administration training

Icatibant is administered subcutaneously and patients are taught self-administration during regular visits to their physician, at the first prescription of this medication [22]. pdC1-INH concentrates are administered by intravenous infusion and patients require more extensive training before feeling comfortable with this administration route. As previously described, in 2010, our center initiated a training program to instruct patients on self-administration of pdC1-INH concentrates by intravenous infusion [21]. The training course consisted of a theory session and a practical session during which participants could practice intravenous injection on a simulator arm. Participants were also instructed on how to behave in case of a laryngeal attack, or if unable to administer treatment, and were invited to keep a diary documenting attack characteristics, treatment administered, time from symptom onset to treatment administration, time from treatment administration to symptom resolution, and other relevant features of home therapy.

### Assessments

Patients were visited at baseline (beginning of observation) and followed up for 6 months, and were interviewed once monthly during a phone call conducted by trained personnel. At baseline, demographic data were collected and recorded in a patient chart designed for the study. Other data recorded in the patient chart were the number of attacks in the previous month, the number of treated attacks, the time from symptom onset to treatment administration, and the time from treatment administration to symptom resolution for each attack. These data were extracted from patients’ diaries. Disease severity was also established. To this purpose we used the general disease severity score developed by Bygum and coworkers [23]. This score ranges from 0 to 10 (10 corresponds to the highest disease severity) and is based on the age at disease onset, number of organs ever affected, and need for long-term prophylaxis (age at onset 0–5 years, 3 points; age at onset 6–10 years, 2 points; age at onset, 11–20 years, 1 point; age at onset > 20 years, 0 points; skin edema ever, 1 point; painful abdominal edema ever, 2 points; laryngeal edema ever, 2 points; other clinical manifestations, 1 point; long-term prophylaxis ever, 1 point). In contrast with other disease severity assessment tools, this score does not consider a specific time frame, and is based on medical records and a patient interview thereby reducing recall bias and subjective interpretation. Compliance with therapy was defined as the proportion of attacks that were treated over the entire observation period. Quality of life was assessed in adults, at the end of the observation, using the disease-specific HAE-QoL questionnaire designed by Prior and colleagues [24]. The questionnaire considers seven domains relevant for quality of life: physical functioning and health (four questions, score range 4–23); disease-related stigma (three questions, score range 3–15); emotional role and social functioning (four questions, score range 4–20); concern about offspring (two questions, score range 2–10); perceived control over illness (four questions, score range 4–20); mental health (four questions, score range 4–24); treatment difficulties (four questions, score range 4–23). The maximum score is 135, and higher scores indicate better quality of life. The HAE-QoL questionnaire, which is validated for use in subjects aged ≥ 18 years, is protected by Spanish intellectual property law and owned by La Fundación para la Investigación Biomédica del Hospital Universitario La Paz (Madrid, Spain). It was kindly made available to us by T. Caballero (Hospital La Paz Institute for Health Research, Madrid, Spain).

### Data analysis

Data were analyzed by descriptive statistics. Means and standard deviations of measured variables and proportions of treated attacks were calculated. A correlation analysis using the Pearson χ^2^ test, with *p* ≤ 0.05 defining statistical significance, was performed to establish whether the choice of treatment strategy (home-based or hospital-based) might correlate with factors including age (pediatric age [<15 years] and adult age [≥15 years]), age at diagnosis, sex, level of education, disease severity score, and total number of attacks over the observation period. The correlation between disease severity score (≥7 [severe disease] or < 7 [mild to moderate disease]) and choice of therapeutic strategy, compliance, and quality of life was also investigated. Statistical analysis was performed using IBM SPSS Statistics software.

## Results

Between August 2014 and January 2015, a total of 62 patients affected by C1-INH-HAE were in treatment at our center. Six patients were lost to follow-up. The remaining 56 (60.7 % female, mean [±SD] age 36 years [±19.6]) were divided into three groups according to treatment received: home-based therapy with pdC1-INH (*n* = 25), home-based therapy with icatibant (*n* = 12), and hospital-based therapy with pdC1-INH (*n* = 19). Characteristics of the study population and treatment groups are summarized in Table 1. Twelve of the 56 patients observed (21.4 %) were aged < 15 years. The mean disease severity score determined according to Bygum et al. [23] was 6.9 (maximum severity score = 10) in the overall population and 7.3, 6.7, and 6.6 in patients receiving home-based pdC1-INH, home-based icatibant, and hospital-based pdC1-INH, respectively; 60.0 %, 58.3 %, and 47.4 % of patients in the three treatment groups, respectively, had severe disease (score ≥ 7). No statistically significant differences were found in the mean disease severity score and in the proportion of patients with severity score ≥ 7 between home-based and hospital-based therapy and between treatment strategies.

**Table 1:** Study population and treatment group characteristics

	Home therapy pdC1-INH (*n* = 25)	Home therapy icatibant (*n* = 12)	Hospital therapy pdC1-INH (*n* = 19)	Overall (*n* = 56)
Sex, *n* (%)				
Female	14 (56.0)	8 (66.7)	12 (63.2)	34 (60.7)
Male	11 (44.0)	4 (33.3)	7 (36.8)	22 (39.3)
Age, yrs, mean, (±SD)	33.0 (19.0)	36.0 (11.5)	39.5 (24.2)	36 (19.6)
Age				
≥ 15 years, *n* (%)	18 (72.0)	12 (100.0)	14 (73.7)	44 (78.6)
< 15 years, *n* (%)	7 (28.0)	0	5 (26.3)	12 (21.4)
Age at diagnosis, yrs, mean, (±SD)	20 (16.0)	30 (12.0)	26 (20.0)	25 (17.0)
Time since diagnosis, yrs, mean, (±SD)	13.0 (8.0)	6.0 (6.0)	13.0 (12.0)	11 (9.0)
Disease severity score, mean, (range)	7.3 (3–10)	6.7 (4–9)	6.6 (3–10)	6.9 (3–10)
Duration of home therapy, months, mean, (range)	25.1 (6–60)	32.7 (12–60)	-	-
Receiving long-term prophylaxis, *n* (%)	6 (24.0)	2 (16.7)	5 (26.3)	13 (23.2)

*Abbreviation definitions*: pdC1-INH plasma-derived C1-esterase inhibitor concentrate, *SD* standard deviation

On average, patients had been on home-based therapy for 25 months with pdC1-INH concentrates and for 33 months with icatibant. Thirteen patients (23.2 %) were using long-term prophylaxis with danazol (*n* = 9), stanazolol (*n* = 1), or C1-INH concentrates (*n* = 3).

**Fig. 1:**
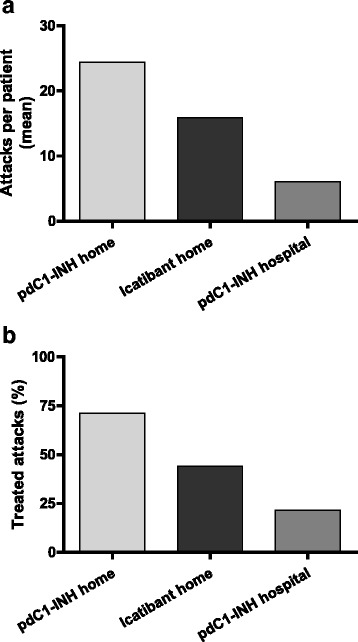
Attack frequency and compliance with treatment in the three treatment groups over 6 months. *Legend* Mean number of attacks per patient (**a**), proportion of attacks treated (compliance) (**b**); pdC1-INH = plasma-derived C1-esterase inhibitor concentrate

During the 6-month observation period, a total of 918 attacks were reported, of which 544 (59.2 %) were treated. Patients treated with pdC1-INH home-based therapy had a total of 611 attacks (mean [±SD] per patient attack number, 24.4 [±26.1] over 6 months), and those treated with icatibant home-based therapy had a total of 191 attacks (mean [±SD] per patient attack number, 15.9 [±12.0] over 6 months): Fig. 1a. Patients receiving hospital-based pdC1-INH therapy had a total of 116 (mean [±SD] per patient attack number, 6.1 [±6.5] over 6 months). The difference in the total number of attacks between home-based therapy (with pdC1-INH and icatibant) and hospital-based therapy was statistically significant (*p* = 0.002, Pearson χ^2^ test).

Patients on home-based therapy had better compliance as suggested by the greater proportion of treated attacks (435/611 [71.2 %] with pdC1-INH and 84/191 [44.0 %] with icatibant), relative to those receiving hospital-based pdC1-INH treatments (25/116 [21.6 %]): Fig. 1b. The difference in the rates of treated attacks between home-based therapy (with pdC1-INH and icatibant) and hospital-based therapy was statistically significant (*p* = 0.003, Pearson χ^2^ test).

The mean times from symptom onset to treatment administration were similar among treatment groups (1.8 [±0.4], 2.6 [±1.1], and 3.1 [±9.7] hours respectively for home-based pdC1-INH, home-based icatibant, and hospital-based pdC1-INH), as were the mean times from treatment administration to symptom resolution (11.3 [±10.2], 10.9 [±9.1], 13.2 [±12.3] hours, respectively). No statistically significant differences were found in the mean times from symptom onset to treatment administration and from treatment administration to symptom resolution between treatment groups.

**Fig. 2:**
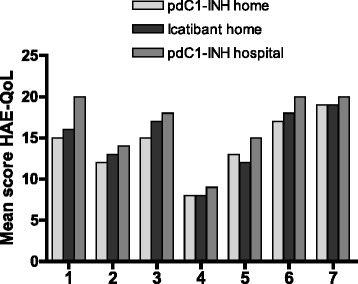
Mean scores for the 7 domains of the hereditary angioedema-quality of life (HAE-QoL) assessment [24], by treatment group. *Legend* (1) Physical functioning and health (4 questions, score range 4–23), (2) Disease-related stigma (3 questions, score range 3–15), (3) Emotional role and social functioning (4 questions, score range 4–20), (4) Concern about offspring (2 questions, score range 2–10), (5) Perceived control over illness (4 questions, score range 4–20), (6) Mental health (4 questions, score range 4–24), (7) Treatment difficulties (4 questions, score range 4–23); pdC1-INH = plasma-derived C1-esterase inhibitor concentrate

The assessment of quality of life in adults using the HAE-QoL [24] administered at the end of observation showed that those with the best quality of life (highest score) were the patients receiving hospital-based pdC1-INH therapy (mean total score 116.7, 75th percentile of healthy age- and sex-matched population), followed by patients receiving home-based therapy with icatibant (102.6, 56th percentile) and pdC1-INH home-based therapy (99.5, 55th percentile): Fig. 2. The difference between treatment groups did not reach statistical significance.

No statistically significant differences were seen between patients receiving home-based therapy or hospital-based therapy, with regard to age (pediatric or adult), age at diagnosis, sex, level of education, and disease severity score (Table 1). The disease severity score was found to correlate significantly (*p* = 0.008) with quality of life, with more patients affected by severe disease (32.2 %) having a worse quality of life (<50th percentile) compared to those with mild to moderate disease (16.7 %) (Table 2). No statistically significant correlation was found between disease severity and treatment strategy (*p* = 0.74) and between disease severity and compliance (*p* = 0.32).

**Table 2:** Therapy preference, compliance, and quality of life in patients stratified according to disease severity

	Treatment strategy		Compliance		Quality of life^a^	
	home *n* = 37	hospital *n* = 19	≥50 % *n* = 27	<50 % *n* = 29	≥50th pct *n* = 29	<50th pct *n* = 11
DSS ≥ 7	22 (70.9 %)	9 (29.1 %)	16 (51.6 %)	15 (48.4 %)	19 (67.8 %)	9 (32.2 %)
DSS < 7	15 (60.0 %)	10 (40.0 %)	11 (44.0 %)	14 (56.0 %)	10 (83.3 %)	2 (16.7 %)

*Abbreviation definitions: DSS* disease severity score, *pct* percentile

^a^ Quality of life was assessed only in patients aged ≥ 18 years (*n* = 40)

## Discussion

This observational study involving 56 patients with C1-INH-HAE who experienced over 900 swelling attacks shows that home-based treatment (with pdC1-INH concentrates or icatibant) was associated with significantly better therapy compliance, compared with hospital-based treatment (with pdC1-INH concentrates). Patients on home-based therapy with pdC1-INH and icatibant treated over 70 % and 40 % of their attacks, respectively, while patients receiving hospital-based care had only about 20 % of their attacks treated. Quite unexpectedly, quality of life showed a tendency to be better in patients receiving hospital-based therapy. Patients on home-based therapy were found to have a significantly greater number of attacks (approximately 4 attacks/month in patients using pdC1-INH and 3 attacks/month in those using icatibant) compared with patients treated in the hospital (approximately 1 attack/month), while no differences were found between patients on home- or hospital-based therapy with regard to sex, age, age at diagnosis, level of education, and disease severity.

The benefits of self-therapy for patients affected by C1-INH-HAE are supported by a large body of evidence [7, 8]. This is reflected in current guidelines for the management of C1-INH-HAE, according to which therapy self-administration should be offered to all patients affected by this condition [17–20]. Recent surveys, and our experience as well, have shown that most patients are willing to learn self-administration, and that self-injection skills are acquired quite rapidly, with only a minority of patients deciding against home therapy after being trained [21, 25–29]. A recent paper discussing strategies to facilitate home-based treatment, has underlined the importance of patient and disease characteristics when evaluating the suitability of patients for training in home therapy [8]. In particular, relevant patient and disease characteristics included mental and physical ability to learn self-injection, patient reliability, patient willingness and motivation, and presence of adequate veins for intravenous administration. Among disease characteristics, high frequency of attacks made home therapy very appropriate. As a rough guideline, the authors suggested that patients who have at least two attacks per month will get the greatest benefit, although highly motivated patients with less frequent attacks may also benefit from home therapy [8]. Patients with a high frequency of attacks are also eligible for long-term prophylaxis with C1-INH concentrates or attenuated androgens, according to current guidelines [18]. The decision to initiate prophylaxis depends however on several other factors, including disease severity, patient quality of life, resource availability, and inadequate symptom control with on-demand therapy [18]. It should also be reminded that breakthrough attacks occur in the majority of patients on prophylaxis, and that on-demand therapy should remain available for these patients [18]. Among our patients, less than one-fourth were on prophylaxis, despite a mean disease severity score close to 7 and indicative of a severe condition. According to our experience, treatments currently available for C1-INH-HAE prophylaxis are refused by some patients because of the inconvenient twice weekly injection schedule (C1-INH-concentrate, Cynrize^®^), and unfavorable side effect profile (attenuated androgens).

In the management of C1-INH-HAE, similarly to a number of other chronic conditions, poor compliance is a major, but often unrecognized problem leading to therapeutic failure, adverse events, and avoidable medical costs [30]. In patients treated at home, adherence to therapy is even more crucial because such patients are not regularly seen by a healthcare professional [28]. According to the results of a recent survey, therapy self-administration is associated with high satisfaction with treatment and good compliance with it [29]. Our findings show a significantly better compliance (higher rate of treated attacks) with treatment in the group receiving home-based therapy, relative to hospital-based therapy. The lower rate of treated attacks in patients referring to a hospital could be explained by the fact that the awareness of rare diseases like C1-INH-HAE among the emergency department personnel is often insufficient resulting in the inadequate treatment of a swelling attack, or no treatment [4, 31]. The compliance of the two groups on home-based therapy was numerically and substantially different, with patients using pdC1-INH concentrates (71 % compliance) being apparently more compliant than those using icatibant (44 % compliance). The reason for this difference is unclear. A longer mean time from diagnosis in patients on pdC1-INH (13 years) vs. patients on icatibant (6 years) could account for this difference, as a longer experience with the management of attacks may be associated with a greater promptness to treat them. In addition, the group treated with icatibant did not include any children because icatibant is not approved for treatment in patients aged <18 years in Italy, which may also account for a decreased tendency to treat any attack. Our data also suggest that the lower the number of attacks per month, the less the tendency to treat the attacks. This, as well, may explain why patients on home-based therapy with icatibant (approximately 3 attacks per month) tend to have lower compliance than patients on home-based C1-INH treatment (more than 4 attacks per month).

In recent years, increasing effort has been devoted to the study of patient-reported outcomes in HAE [32], including the quality of life [24, 33]. Clinical outcomes such as the severity and the frequency of attacks are often insufficient to describe the full impact of HAE on patients’ lives. The considerable burden of the disease and its negative effect on patient quality of life is well documented [24, 34–38]. A number of studies have also investigated the impact of the switch to self-administered therapy on the quality of life. One of these studies assessed the quality of life using the Dermatology Life Quality Index (DLQI) and the 36-Item Short Form (SF-36) Health Survey questionnaires (neither of which is validated for HAE) in seven patients switching to home therapy [38]. The DLQI score improved in a statistically and clinically significant way, and the SF-36 showed substantial improvements in all items (physical functioning, social functioning, physical role functioning, emotional role functioning, mental health, vitality, bodily pain, general health). A later study by the same authors investigated the changes in the burden of illness before and after the switch to home therapy using a questionnaire that addressed various aspects of the HAE burden, namely the psychological impact, the impact on physical activities, the worry about suffocation, the concern about heredity, and the fear of treatment side effects [7]. The switch was associated with a substantial and statistically significant improvement in all five points considered. Although the overall picture emerging from these studies is that the switch to home therapy is associated with an improvement in quality of life, there is a need for trials assessing this outcome with tools specific for HAE. The present study used a validated disease-specific questionnaire for use in patients with HAE [24]. Our findings show, in contrast with most of the available evidence, a trend towards lower quality of life in patients on home-based treatment compared with those treated in the hospital. A possible explanation is that our patients on home therapy had a significantly higher frequency of attacks and, likely, a more severe condition impacting negatively on their quality of life. The comparison of outcomes in patients stratified according to the disease severity index, showed indeed a significant correlation between severe disease (index ≥ 7) and reduced quality of life. No difference in quality of life, and a worsening in some domains, has also been reported by a recent study [39]. This study, which used the SF-36 questionnaire (version 2), found a similar quality of life of self-injecting and non-self-injecting patients, with the exception of the domain “general health”, which was significantly worse in patients on self-therapy [39]. According to the authors, this finding could be explained by the fact that home-based therapy, despite a number of benefits, may constantly remind patients of their chronic condition. An association between high attack frequency and low quality of life was reported also in a study using the patient-completed EuroQol 5 Dimensions 5 Levels (EQ5D-5L) questionnaire that evaluates health based on five dimensions, including mobility, self-care, usual activities, pain/discomfort, and anxiety/depression [37]. Patients completed the questionnaire both for their current health state and the state during their last HAE attack. Patients with an attack frequency > 30 attacks per year were found to have a significantly lower perceived current health state compared to those with less frequent attacks.

The significantly higher frequency of attacks in our patients on home therapy compared with those treated in the hospital suggests that this disease characteristic may be predictive of a preference for home-based treatment. Of note, the stratification of patients according to disease severity score (<7 or ≥ 7) did not reveal any significant correlation with the use of home- or hospital-based therapy suggesting that disease severity does not influence the choice of the treatment strategy. Furthermore, no significant correlation was found between treatment choice and other patient characteristics, including sex, age, age at disease onset, and the level of education and age. With regard to age, we expected that young age would correlate with increased willingness to learn and use self-therapy. We also expected that a longer time since diagnosis would correlate with reduced willingness to switch from hospital-based therapy to home-based therapy, but this expectation was not confirmed by our findings either. Notably, a recent update of the Icatibant Outcome Survey (IOS) comparing icatibant self-administration vs. administration by health care professionals failed to demonstrate that factors including sex, use of long-term therapy, and attack location are predictive of the preference for self-administration [13].

The time from symptom onset and to therapy administration was not significantly different between home-based and hospital-based treatment, though there was a trend towards a more rapid administration in patients treated at home (1.8 h for patients in treatment with pdC1-INH at home vs. 3.1 h for patients treated with pdC1-INH in hospital). Most data from the literature show statistically significant improvements in time to treatment after the switch from hospital to home therapy [11, 13, 15, 38]. The reason why this variable did not improve significantly in our patients is currently unclear. The time from therapy administration to attack resolution was not significantly different either between the two treatment strategies; it was slightly longer (>10 h for both home and hospital therapy) than most values reported in the literature [9, 11, 40]. The abovementioned update of the IOS comparing icatibant self-administration vs. administration by health care professionals did not find either any significant difference between the two treatment strategies with regard to attack duration and time to resolution [13]. According to the authors this may be explained by an improvement (shortening) in times to treatment also in patients treated by healthcare professionals, a possible consequence of the increased compliance with current guidelines that recommend to treat an attack as early as possible.

The fact that variables including the number of attacks and treated attacks, the time from symptom onset to treatment administration, and the time from treatment administration to symptom resolution were mostly based on patient recollection and entry in their diaries may have introduced some bias in the present study. The lack of a severity assessment for each attack over the 6 months of observation, as well as the lack of information about the body site affected, is another potential limitation, as these variables, along with frequency, may also influence the decision between hospital- and home-based treatment. At the time of our observation there were no generally accepted patient-reported outcome tools such as, for example, the questionnaire for the assessment of HAE recently developed by Bonner et al. within the IOS [32]. We assessed instead the general severity of disease based on patient medical records and recollection using the severity scoring system developed by Bygum et al., which takes into account objective variables like age of disease onset, body areas affected, and need for long-term prophylaxis [23]. The observational design and the small size of the analyzed population are other limitations of our study. The latter limitation is hard to avoid, as C1-INH-HAE is a rare disease and the recruitment of sufficient patient numbers is extremely difficult. Despite these limitations, the present study describes real-life C1-INH-HAE patients using different strategies for the treatment of acute attacks and may provide useful information with regard to patient subgroups most likely to benefit from self-administration.

## Conclusion

Frequency of attack appears as an important factor in the choice between home-based therapy and hospital-based therapy. The use of home-based therapy with both pdC1-INH and icatibant was found to correlate with high attack frequency. Compared with hospital-based therapy, home-based therapy with both medications was associated with significantly better adherence to treatment, as measured by the proportion of treated attacks, while no difference was seen in patient quality of life. Treatment at home should be offered to all patients with C1-INH-HAE and especially to those with a high frequency of attacks.

## Abbreviations

C1-INH: C1-esterase inhibitor
C1-INH-HAE: Hereditary angioedema with C1-inhibitor deficiency
DLQI: Dermatology life quality index
DSS: Disease severity score
EQ5D-5L: EuroQol 5 Dimensions 5 Levels
HAE: Hereditary angioedema
IOS: Icatibant outcome survey
pd: Plasma-derived
QoL: Quality of life
SD: Standard deviation
SF-36: 36-Item short form
